# Comprehensive Grading System for Experimental Autoimmune Uveitis in Mice

**DOI:** 10.3390/biomedicines11072022

**Published:** 2023-07-18

**Authors:** Avik Shome, Odunayo O. Mugisho, Rachael L. Niederer, Ilva D. Rupenthal

**Affiliations:** 1 Buchanan Ocular Therapeutics Unit, Department of Ophthalmology, The New Zealand National Eye Centre, University of Auckland, Auckland 1142, New Zealand; a.shome@auckland.ac.nz (A.S.); lola.mugisho@auckland.ac.nz (O.O.M.); 2 Department of Ophthalmology, The New Zealand National Eye Centre, University of Auckland, Auckland 1142, New Zealand; r.niederer@auckland.ac.nz; 3 Te Whatu Ora Te Toka Tumai, Auckland 1142, New Zealand

**Keywords:** uveitis, inflammation, experimental autoimmune uveitis, fundus imaging, optical coherence tomography

## Abstract

Experimental autoimmune uveitis (EAU) is the most commonly used animal model to study the progression of chronic uveitis and to test various therapies to treat the disease. However, to accurately evaluate the effectiveness of such treatments, a grading system that combines the latest imaging techniques with definitive quantitative grading thresholds is required. This study aimed to develop a comprehensive grading system that objectively evaluates EAU progression in C57BL/6J mice. EAU was induced following immunisation with interphotoreceptor retinoid-binding protein (IRBP) and pertussis toxin. Weekly fundus and optical coherence tomography (OCT) images were acquired over 12 weeks using a Micron IV imaging system. Each mouse was graded (between 0 to 4) based on changes seen on both the fundus (optic disc, retinal blood vessels and retinal tissue) and OCT (vitreous and retinal layers) images. A total EAU response (with a maximum score of 48) was calculated for each mouse based on the sum of the individual scores each week. Analysis of the clinical scores depicted a gradual increase in inflammatory signs including optic disc and vascular swelling, leukocyte infiltration in the vitreous, lesions in the retina and formation of granulomas and hyper-reflective foci in the retinal layers in EAU mice, with most signs reaching a plateau towards the end of the study period. Development of these signs into sight-threatening complications such as optic disc atrophy, structural damage to the retina and subretinal oedema were noted in 80–90% of mice suggesting consistent disease induction. Overall, a comprehensive and objective grading system encompassing all pathologies occurring in EAU mice was developed to enhance the preclinical evaluation of novel uveitis treatments.

## 1. Introduction

Animal models of uveitis have been established in a variety of species investigating various aspects including mechanisms of disease progression, aetiology and novel therapies [1]. While earlier studies were often conducted in larger animals such as rabbits and guinea pigs [2] which are now mainly used to study specific aspects of uveitis only, rodents have been the most widely used model due to lower associated costs and availability of well-characterised inbred lines [3]. Another important consideration when choosing a suitable animal model, especially when investigating novel therapies, is the antigen response that triggers inflammation. For instance, an endotoxin-induced uveitis (EIU) model, using lipopolysaccharide (LPS) as the stimulant, may be favourable for short-term studies as inflammation is usually resolved within 72 h [4]. On the other hand, an experimental autoimmune uveitis (EAU) model, established using various eye-specific antigens, such as interphotoreceptor retinoid-binding protein (IRBP) or retinal soluble antigen (S-Ag), might be more useful to study aspects of uveitis associated with chronic manifestations of the disease, with inflammation generally lasting several weeks and causing more structural damage to the eye [5]. In humans, non-infectious uveitis represents the most challenging form of uveitis as it is difficult to treat and can be associated with systemic autoimmune conditions such as juvenile idiopathic arthritis or ocular autoimmune diseases such as birdshot chorioretinopathy [6,7]. Non-infectious uveitis often involves multiple relapses, and requires long-term therapy with corticosteroids, overuse of which can lead to side effects such as cataract formation and glaucoma [8]. Although alternative methods of treatment for non-infectious uveitis exist, such as T lymphocyte inhibitors (e.g., methotrexate) and TNFα inhibitors (e.g., adalimumab), they may be more difficult to access and carry their own risks and side effects [6]. Therefore, development of alternative therapies for the treatment of non-infectious uveitis in humans with decreased side effects remains a priority with EAU rodent models identified as the preferred tool for such preclinical investigations [6].

EAU rodent models have been used since the 1970s to study various inflammatory pathways involved in the manifestation of chronic uveitis [9,10]. In earlier studies, disease progression was only graded using histological scoring [10,11], which was later followed by fundoscopy facilitating the real-time tracking of uveitis progression [12]. With advancements in microscopic techniques, topical endoscopy fundus imaging (TEFI) was later used to provide high-resolution images of the mouse fundus [13], which further aided the development of a grading system to better track uveitis progression [14]. For example, Xu et al. [14] developed an EAU grading system in a mouse model describing fundus changes with regards to optic disc, retinal vessels, and retinal tissue at various stages of the disease. The study noted the development of swelling and lesions in the optic disc, swelling of retinal blood vessels, presence of perivascular sheathing (cuffing) of vessels and manifestation of lesions and scarring in retinal tissues. Although detailed, it is important to note that changes observed in the fundus are only part of the sight-threatening pathological signs associated with uveitis. For a better understanding of the effects of ocular inflammation, optical coherence tomography (OCT) has also been used to obtain high-resolution cross-sectional in vivo real-time images of ocular tissues, in particular the retina [15]. OCT is a useful adjunct to the macroscopic visualisation of the fundus, and can detect more subtle pathology, which nonetheless can still be vision threatening [6]. Harimoto et al. [16] developed a grading system using spectral domain OCT (SD-OCT) imaging that complements clinical fundus scoring, offering a snapshot of the overall ocular health at any given time. However, such grading systems from fundus images and OCT have seldom been used in combination in preclinical studies. This is partly due to a lack of access to both imaging technologies and partly due to the shortfall of OCT grading systems in providing detailed definitive grading thresholds, ultimately resulting in ambiguity of the clinical scores assigned by multiple observers. 

With recent improvements and availability of preclinical imaging technologies such as the Micron III and IV (Phoenix Research Labs; Pleasanton, CA, USA) and the iVivio LAB OCT (OcuScience, Henderson, NV, USA), high-resolution images, both fundus and OCT, can be obtained in rodents using the same machine [17,18,19]. Therefore, with the combination of both technologies, there is a need for an integrated grading system for assessing complete ocular health and disease states in EAU rodent models. Using a Micron IV imaging platform, we obtained high-resolution fundus images of the optic disc, retinal blood vessels and retinal tissues along with OCT images of the retinal layers and vitreous to establish a comprehensive grading system that integrates clinical inflammation scores from the morphological changes seen (from here on referred to as inflammatory elements). This enables the more accurate tracking of uveitis development and progression, integrating clinical scores from both technologies. While fundus images provide information about retinal blood vessel and optic disc health, certain crucial inflammatory signs in early stages of the disease, such as immune cells in the vitreous, are often difficult to visualise in the fundus alone [14], but are clearly visible on OCT images [20]. Therefore, an integration of both technologies is required. In addition, a grading system that combines the strengths of two imaging technologies, fundoscopy and OCT, is better able to attain the sensitivity and specificity required to objectively test the efficacy of novel anti-inflammatory uveitis therapies. We tracked uveitis development over a 12-week period, longer than Harimoto and colleagues (only 4–5 weeks) [16], to cover the entire inflammatory sign spectrum, observing the progression of sight-threatening complications in the retinal layers and vitreous using OCT images in conjunction with signs observed in fundus images. Overall, the aim of the present study was to develop a comprehensive grading system of all pathologies in EAU mice using a combination of fundus and OCT imaging to offer a more robust preclinical assessment of novel uveitis therapies in the EAU mouse model.

## 2. Materials and Methods

### 2.1. Animals

Six- to eight-week-old female C57BL/6J mice obtained from Jackson Laboratory (Bar Harbor, ME, USA) were used in this study. Animals were bred by the Vernon Jenson Unit, University of Auckland, New Zealand, in a specific pathogen-free containment facility. Mice were housed in groups of five animals per cage with corncob bedding material under normal cyclic light conditions (12 h light; 12 h dark) with food and water ad libitum. Mice were fed regular chow (Teklad Global 18% protein rodent diet, Harlan Laboratories, Madison, WI, USA). All procedures were carried out in accordance with local legislations approved by the University of Auckland Animal Ethics Committee (AEC2882 approved on 9 November 2020) and in accordance with the Association for Research in Vision and Ophthalmology (ARVO) resolution and the ARRIVE guidelines for use of animals in research [21]. Experiments were conducted in anaesthetised mice using intraperitoneal (i.p.) injection of ketamine (PhoenixPharm, Auckland, New Zealand; 50 mg/kg) and medetomidine (Domitor^®^, Zoetis, Rhodes, NSW, Australia; 0.5 mg/kg). Following baseline ocular assessments and EAU induction, anaesthesia was reversed by i.p. injection of atipamezole (Antisedan^®^, Zoetis, Rhodes, NSW, Australia; 5 mg/kg). EAU was induced in randomly assigned mice (6 mice; 12 eyes), while control mice (4 mice; 8 eyes) received an equivalent injection of saline only.

### 2.2. EAU Induction

**Figure A1:**
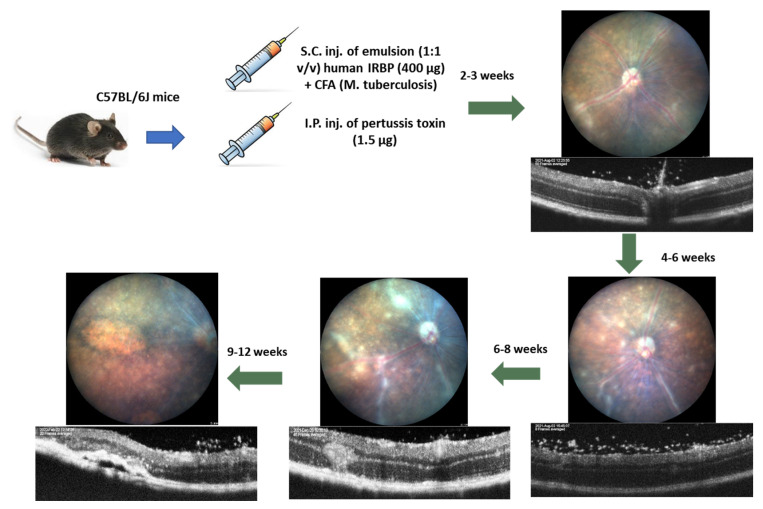
Schematic representation of the experimental design. C57BL/6J mice received a subcutaneously (S.C.) injection (inj.) of 400 µg of human IRBP_1–20_ emulsified in CFA at a 1:1 volume/volume ratio. Mice were concurrently injected with 1.5 µg of pertussis toxin intraperitoneally (I.P.). Uveitis signs were monitored weekly using fundus and OCT imaging. An increased severity of ocular inflammatory signs was noted in weeks 2–12. Mice were euthanised for tissue collection at the end of the 12–week study period.

EAU was induced as previously described [3,16,22]. Briefly, mice were immunised by subcutaneous (s.c.) injection (23G needle) of 150 µL of a 1:1 emulsion containing 400 μg human IRBP peptide 1–20 (IRBP_1–20_; GPTHLFQPSLVLDMAKVLLD; China Peptides, Shanghai, China) in complete Freund’s adjuvant (CFA; Sigma-Aldrich, St Louis, MO, USA) supplemented with Mycobacterium tuberculosis H37RA (2.5 mg/mL; Difco Laboratories, Detroit, MI, USA), distributed between the base of the tail and the right and left flank (50 µL at each site) (Figure A1). The emulsion was sonicated for 5 min at room temperature to ensure uniform distribution of the oil droplets in the water phase resulting in a highly viscous mixture. Sites of injection were then wiped with distilled water and ethanol to prevent any skin reactions to potential emulsion backflow. Mice received a concurrent i.p. injection of 1.5 µg of pertussis toxin (PTX, Enzo Life Sciences, Farmingdale, YN, USA) in 50 µL of saline.

### 2.3. Fundus Imaging and Grading

**Figure 1:**
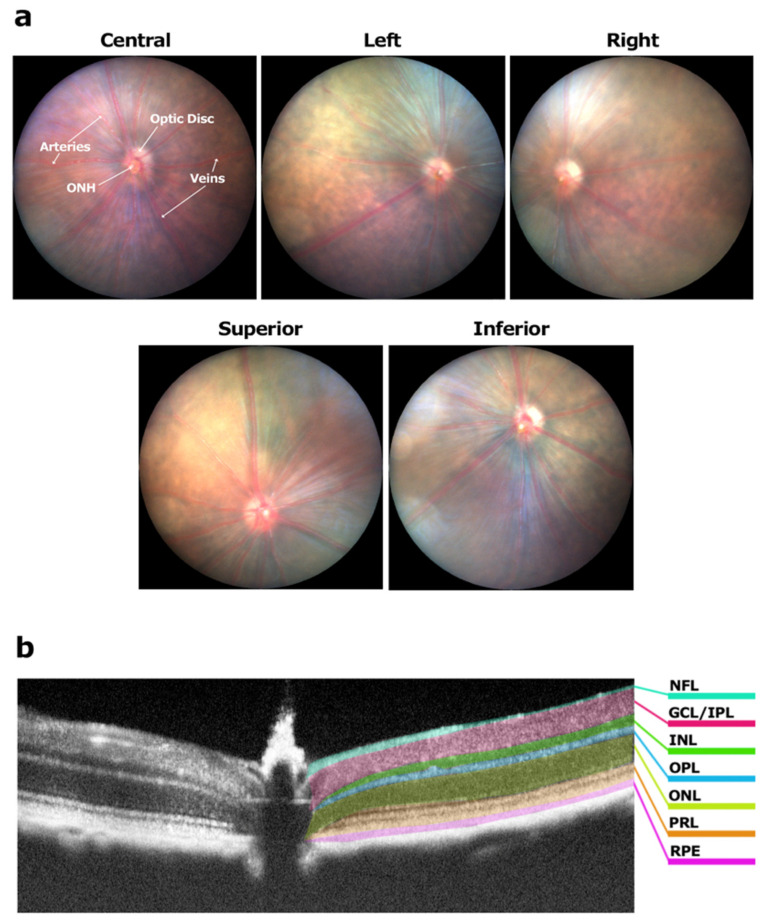
Orientation of a set of five fundus images and retinal layers depicted in an OCT image. (**a**) Fundus images used for grading with an image having the ONH in the central position (depicting the position of optic disc, ONH, arteries and veins), the left side of the eye (ONH is oriented to the right of the image), the right side of the eye (ONH is oriented to left of the image), image with ONH oriented towards the bottom of the fundus image exposing more of the superior region and oriented towards the top of the fundus image exposing more of the inferior region of the eye. (**b**) For OCT image collection, each eye was oriented in the above positions to expose the peripheral regions of the eye and the entire surface of the eye was scanned using fundus image guiding. Retinal layers were clearly distinguishable on the OCT image. NFL: nerve fiber layer; GCL: ganglion cell layer; IPL: inner plexiform layer; INL: inner nuclear layer; OPL: outer plexiform layer; ONL: outer nuclear layer; PRL: photoreceptor layer; RPE: retinal pigment epithelium.

Following pupil dilation using 1% tropicamide solution (Minims, Bausch + Lomb, Surrey, UK), mice were placed on a heated platform to regulate their body temperature and prevent cold-induced cataracts. The cornea was kept moist using a lubricating eye gel (Polygel^®^; Alcon Laboratories, Frenchs Forrest, NSW, Australia). High-resolution fundus images were acquired using a Micron IV imaging system (Phoenix Research Labs, Pleasanton, CA, USA) at baseline prior to EAU induction and weekly thereafter for a period of 12 weeks. A set of five fundus images was collected for each eye (Figure 1a); one with the optic nerve head (ONH) positioned in the centre, one each with the ONH oriented towards the left and right side and two with the ONH oriented towards the bottom (exposing the superior region) and top (exposing the inferior region). From the fundus images, the condition of the optic disc, retinal blood vessels, retinal tissues and structural damage were assessed using the following protocol. All assessments were performed by an unmasked researcher with results validated by a masked observer.

#### 2.3.1. Optic Disc

Using the image with the central orientation of the ONH (Figure 1a), the area of the optic disc covered in lesions was measured using ImageJ software version 1.46r (National Institutes of Health, Bethesda, MD, USA). To quantify the blurriness of the optic disc, the proportion if visible blood vessels within the optic disc was quantified and a weekly grade for optic disc condition was assigned.

#### 2.3.2. Retinal Blood Vessels

Using all five fundus images, retinal blood vessel health was assessed based on the presence of vessel swelling, perivascular sheathing or cuffing and visibility of blood vessels. Blood vessels in a fundus image can be either arteries or veins, the former having a smaller diameter and a broken white line running through the middle (Figure 1a), while veins are generally larger in diameter without the white middle line. The presence of swelling was noted in one or more blood vessels to determine a grade. The diameter of blood vessels and cuffing was measured using the ‘draw line’ and ‘measure’ functions in ImageJ to assign a grade.

#### 2.3.3. Retinal Tissue

Using all five fundus images, retinal tissue health was assessed by counting the number of round retinal lesions (small white spots) or linear lesions (white lines). Each fundus image was divided into four quadrants and the number of retinal lesions was counted in each quadrant. The area of scaring was determined using the ‘area tool’ in ImageJ.

#### 2.3.4. Structural Damage

For grading structural damage, the numbers of linear lesions and retinal scars were counted. The area of scarring was determined using the ‘area tool’ in ImageJ. The area of the entire eyeball depicted in each of the five fundus images (Figure 1a) was summed to obtain the total surface area of the eye. Similarly, the area of scarring (multiple regions) in each of the fundus images was measured and the total area of scarring was calculated by summation of all regions. The total area of scarred regions was then compared to the total surface area of the eye to calculate the percentage of scarring. Any lesion overlap was normalised by the fact that there was also an overlap in total surface area of the eye depicted in the five fundus images.

#### 2.3.5. Grading

Each mouse eye was graded weekly using the grading system laid out in Table 1 with the help of reference images. The grading system specified both quantitative and qualitative thresholds for each grade with an absolute score between 0 and 4 and without fraction scores. Scores from the left and right eye were then summed to obtain the clinical score for each mouse with the maximal possible score being 8 per inflammatory element assessed. An average score was then calculated for EAU (*n* = 6) and control (*n* = 4) mice. The mean scores along with the standard deviation (depicted by error bars) was then plotted against time to track the progression of each inflammatory element over the 12-week period.

### 2.4. OCT Imaging and Grading

Fundus image-guided OCT images were collected using a Micron IV imaging system in anaesthetised animals as previously described [24]. Each OCT image is a transverse cross-sectional image of the retina (Figure 1b) and vitreous with its position reflected by a red line in the corresponding fundus image. Each eye was scanned completely by moving the transverse scanning beam at set ‘steps’ (each step being 3 µm) along the longitudinal plane of the eye (top to bottom in a fundus image). Each eye was placed in the five fundus positions highlighted in Figure 1a and the entire exposed surface area of the fundus was scanned to collect multiple OCT images. A set of 18 OCT images (nine with the vitreous and nine with retinal layers in focus) was then selected from the captured images for each eye; one at the ONH (centre), four at superior and four at inferior positions. Image selection was based on the number and/or severity of the pathologies. A grade was determined for each eye based on the pathologies depicted in the vitreous and retinal layers using the following protocol.

#### 2.4.1. Vitreous

The presence of white spots in vitreous-focused images was noted including the position (inferior or superior) of the OCT in the corresponding fundus image. The total number of spots was counted in all nine OCT images to assign an overall grade.

#### 2.4.2. Retinal Layers

Retina-focused OCT images were scanned for the presence of hyper-reflective foci (HRF), granulomas, sub-retinal fluid accumulation and disruption of retinal layers as discussed previously [16,25]. A clinical score was determined based on the number of HRF and granulomas as well as the appearance of the retinal layers (e.g., disruption). The score was also based on the severity and size of granulomas and HRF disrupting multiple retinal layers and HRF leading to sub-retinal fluid accumulation.

#### 2.4.3. Grading

Each mouse eye was graded weekly using the grading system laid out in Table 2 with the help of reference images. Each eye was graded with an absolute score between 0 and 4 and the left and right scores were summed to obtain a clinical score for vitreous and retinal layers per mouse per week with a maximum possible score of 8. The clinical scores of all mice in each group were then averaged to obtain a mean score which was plotted against time.

### 2.5. Statistical Analysis

All statistical analyses were conducted using MS Excel 2021 and GraphPad Prism software version 5 (GraphPad Software, San Diego, CA, USA). Clinical scores were presented as mean ± standard deviation. Clinical scores were tested using a multiple *t*-test (with Holm–Sidak post hoc multiple comparison test) to determine significant differences between EAU and control mice. A *p*-value of <0.05 was considered to be statistically significant. The rate of progression for each inflammatory element was calculated from clinical scores relative to those assigned at the first appearance of the sign.

## 3. Results

### 3.1. Fundus Imaging and Grading

High-resolution fundus images showed the optic disc located in the centre of the field of view with 6–12 blood vessels (veins and arteries) radiating from the optic disc (Figure 1a). Retinal tissue seen in between the blood vessels was also clearly visible. No changes were seen in the fundus images of control mice throughout the study period, resulting in a clinical score of 0 for all four inflammatory elements (Table 1). Scoring of the EAU-induced mice is described in more detail below.

#### 3.1.1. Steady Rise in Swelling and Lesions in the Optic Disc Resulted in the Highest Clinical Score by Week 12

**Figure 2:**
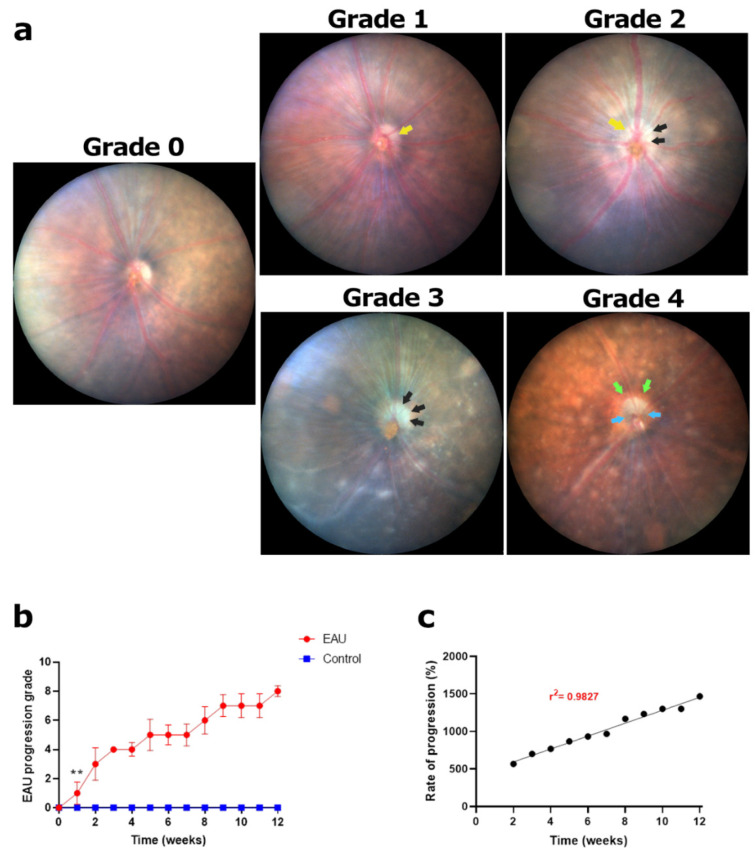
Signs of EAU and clinical grading in the optic disc using fundus images. (**a**) Normal optic disc before inoculation; **grade 0**. Onset of EAU denoted by optic disc swelling; **grade 1**. Yellow arrows indicate optic disc margins. Optic disc swelling followed by formation of lesions and blurry retinal blood vessels within the optic disc margins; **grade 2**. Black arrows indicate lesions in the optic disc. Lesions covering 10–30% of the optic disc and <30% of retinal vessels blurry due to lesions; **grade 3**. ONH atrophy denoted by ‘sunset’ halo and glassy opaque appearance of the optic disc; **grade 4**. Green arrows indicate ‘sunset’ halo around an atrophied ONH and blue arrows indicate a ‘glassy opaque’ appearance of the atrophied optic disc and ONH. (**b**) Each eye was graded based on the pathologies observed in a set of five fundus images collected weekly for a period of 12 weeks. A weekly clinical score for optic disc health (maximum possible score of 8) was calculated for each mouse by adding the scores for the left and right eye. An average weekly score for each mouse in the EAU (*n* = 6 mice) and control (*n* = 4 mice) group along with the standard deviation (error bars) was calculated and plotted against time. Progression of inflammation in the optic disc, tracked over 12 weeks, showed a steady increase in the clinical scores with periods of plateau giving rise to a step-like pattern. Significant differences between EAU and control group clinical scores were calculated using a multiple *t*-test. EAU mice had significantly higher clinical scores from week 1 (** *p* < 0.05) onwards with control mice showing no signs of swelling, lesions, or atrophy in the optic disc throughout the 12-week period. (**c**) Progression of clinical scores in weeks 2–12 relative to the onset of optic disc changes in week 1. The progression was found to be linear (r^2^ = 0.9827) highlighting a steady increase in disease severity.

**Figure A2:**
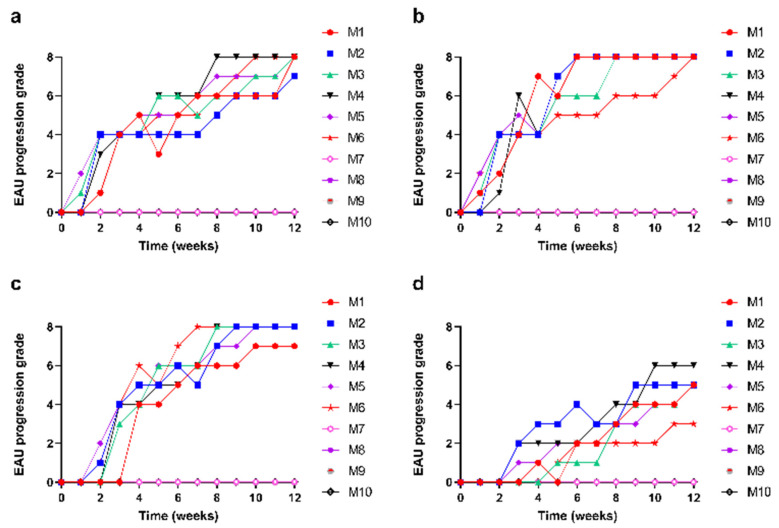
Clinical grading for each inflammatory element (i.e., optic disc, retinal blood vessels, retinal tissue and structural damage) in each mouse using fundus images. Each eye was graded based on the pathologies observed in a set of five fundus images collected weekly for a period of 12 weeks. A weekly clinical score for each inflammatory element (maximum possible score of 8) was calculated for each mouse by adding the scores for the left and right eye. The clinical scores for each mouse in the EAU (M1–M6) and control (M7–M10) group were plotted against time. (**a**) Progression of inflammation in the optic disc showed variability in clinical scores with periods of plateau giving rise to a step-like pattern for most EAU mice (M1–M6). (**b**) Progression of inflammation in retinal blood vessels showed a sharp increase in the first 3–4 weeks followed by a steady rise, reaching the maximum possible score for M1, M2, M4 and M5 by week 6 and M3 and M6 by week 8 and 12, respectively. M6 had an overall slower progression of inflammation in retinal blood vessels. (**c**) Progression of inflammation in retinal tissue showed a sharp increase in weeks 2–4 followed by a step-like growth before reaching the maximum possible score in weeks 7–9 for most mice. M6 did not show any signs of inflammation in retinal tissues within the first 3 weeks but there was a sudden increase in week 4, although the maximum possible score was not reached by the end of the observation period for this mouse. (**d**) Progression of structural damage caused by inflammation showed a step-like increase throughout the study period for most EAU mice with the exception of M6 in which the progression was slow with no response for the first 6 weeks and only a moderate response observed by the end of the study. Most EAU mice (M1–M5) showed some form of structural damage due to inflammation; however, the maximum possible score was not reached by any of the mice indicating that none had any retinal atrophy. Control mice showed no signs of inflammation in optic disc, retinal blood vessels, retinal tissue or any structural damage to the retina throughout the 12-week study period.

Detailed examination of the fundus images showed increased optic disc margin size (swelling), reduced visibility of retinal vessels connecting to the ONH, presence of lesions within the optic disc margins and overall optic disc health (Table 1, Figure 2a). In the initial stages (weeks 2–4) of the disease, optic disc swelling was the first sign to appear in all EAU mice (Figure 2a; yellow arrows; grade 1), while blood vessels within the optic disc margin were still clearly visible with no lesions. Optic disc swelling was not uniform across all EAU mice with some showing a delayed response initiated in week 3 (Figure A2a). As the disease progressed (weeks 5–7), blood vessels and optic disc margins became blurry, with areas of the optic disc covered in lesions resulting in a reduced visibility of the blood vessels (Figure 2a, grade 2–3). The development of lesions occurred at a variable pace which was reflected by a step-like progression of the clinical scores, with periods of steady growth interspersed by periods of plateau in weeks 5–12 (Figure 2b). In the latter stages of the disease (weeks 8–12), swelling subsided while the area covered by the lesions increased. Formation of a ‘sunset’ halo around the optic disc (Figure 2a; green arrows) and a glassy opaque appearance (Figure 2a; blue arrows) within the optic disc suggested the onset of optic nerve atrophy (grade 4). While optic nerve atrophy was not observed in all EAU mice, 80–85% had extensive lesion coverage (Section 3.5), reaching the highest possible score by week 12 (Figure 2b, grade 4). Clinical scores in EAU mice were significantly higher than those of control mice from week 1 onwards (Figure 2b, ** *p* value < 0.05). Overall, EAU signs in the optic disc occurred at a steady rate confirmed by the linear progression of clinical scores over the 12-week period (Figure 2c).

#### 3.1.2. Rapid Progression of Cuffing in Retinal Blood Vessels Resulted in the Highest Clinical Score by Week 8

**Figure 3:**
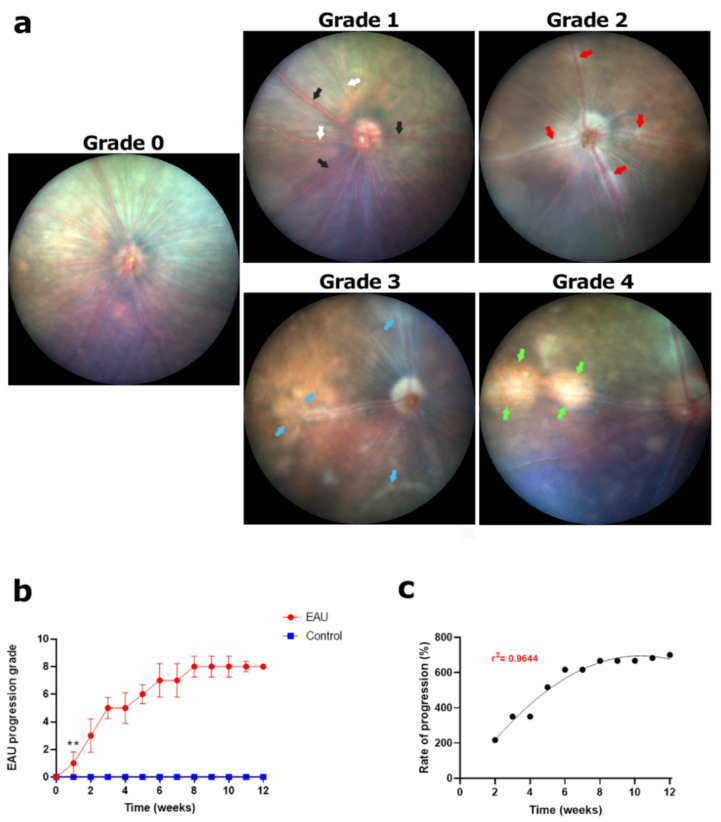
Signs of EAU and clinical grading in retinal blood vessels using fundus images. (**a**) Normal retinal blood vessels before inoculation; **grade 0**. Swelling of blood vessels (mainly veins); **grade 1**. White arrows indicate arteries and black arrows indicate veins. Formation of a ‘hazy sheath’ or ‘cuffing’ (red arrows) around blood vessels due to presence of perivascular infiltrates (tiny white spots). Thickness of cuffing less than thickness of blood vessel; **grade 2**. Perivascular lesions clumping together to form larger areas of cuffing (blue arrows). Thickness of cuffing greater than thickness of blood vessel; **grade 3**. Large areas of scarring and tissue damage (green arrows) obscuring blood vessels; **grade 4**. (**b**) Each eye was graded based on the pathologies observed in a set of five fundus images collected weekly for a period of 12 weeks. A weekly clinical score for retinal blood vessel health (maximum possible score of 8) was calculated for each mouse by adding the scores for left and right eye. An average weekly score for each mouse in the EAU (*n* = 6 mice) and control group (*n* = 4 mice) along with the standard deviation (error bars) was calculated and plotted against time. Signs of EAU in retinal blood vessels showed a sharp increase in the first 3 weeks followed by a steady growth, reaching the maximum possible score by week 8. Significant differences between EAU and control group clinical scores were calculated using a multiple *t*-test. EAU mice had significantly higher clinical scores (** *p* < 0.05) from week 1 onwards. Control mice showed no signs of blood vessel swelling, cuffing or scarring in blood vessels throughout the 12-week period. (**c**) Progression of clinical scores in weeks 2–12 relative to the onset of changes in retinal blood vessels in week 1. The progression was found to be sigmoidal (r^2^ = 0.9644) highlighting a rapid increase in signs in the early stages of the disease which began to slow down from week 7, reaching a plateau towards the end of the study.

Swelling or engorgement of retinal blood vessels was observed in 50% of EAU mice eyes within the first week post immunisation (grade 1; Figure A2b). Swelling was less prominent in arteries (grade 1; Figure 3a; white arrows) but primarily affected veins (Figure 3a; black arrows) and was found to be resolved in the latter stages of the disease (by weeks 6–8). In weeks 2–5, we noted perivascular infiltrates resulting in higher clinical scores according to Table 1. These infiltrates, appearing as small whitish lesions, formed a ‘hazy sheath’ or ‘cuffing’ around the blood vessels (Figure 3a; red arrows; grade 2). By weeks 5–7, these lesions had clumped together to form larger areas of cuffing around multiple blood vessels (Figure 3a; blue arrows; grade 3), giving the blood vessels a blurry appearance. In the latter stages of the disease (weeks 8–12), we noted large areas of perivascular cuffing leading to scar formation and tissue damage (Figure 3a; green arrows; grade 4). By weeks 8–9, 95–100% of the EAU mice showed signs of extensive cuffing and scarring (Section 3.5) obscuring multiple blood vessels at several segments thereby attaining the highest possible clinical score of 8 (Figure 3b). Thereafter, clinical scores plateaued in weeks 10–12. As expected, clinical scores in EAU mice were found to be significantly higher than those of control mice from week 1 onwards (Figure 3b, ** *p* < 0.05). As evident from the clinical score progression (Figure 3c), EAU signs in retinal blood vessels developed rapidly during the first six weeks followed by a steady progression before plateauing in weeks 10–12. We also noted that areas of perivascular cuffing and scarring correlated with the formation of granulomas, HRF and subretinal fluid as further described below.

#### 3.1.3. Increasing Retinal Tissue Lesions and Scarring Resulted in the Highest Clinical Scores by Week 9

**Figure 4:**
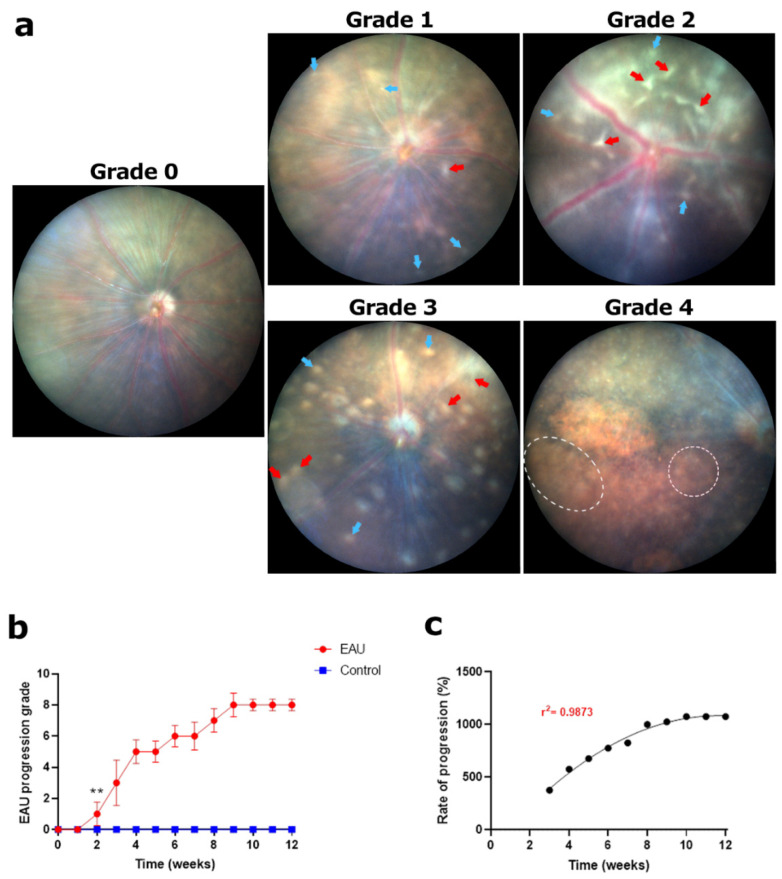
Signs of EAU and clinical grading in retinal tissue using fundus images. (**a**) Normal retinal tissue before inoculation; **grade 0**. Onset of EAU with small round lesions (3–10) (blue arrows) and linear lesions (1–4) (red arrows); **grade 1**. Increased number of round lesions (11–30) and/or linear lesions (5–10); **grade 2**. Increased number of round lesions (>30) and/or linear lesions (>10) and/or small area of scaring (<10%); **grade 3**. Large areas of scarring (white dotted lines) (>10%) and/or retinal atrophy; **grade 4**. (**b**) Each eye was graded based on the pathologies observed in a set of five fundus images collected weekly for a period of 12 weeks. A weekly clinical score for retinal tissue health (maximum possible score of 8) for each mouse was calculated by adding the scores for the left and right eye. An average weekly score for each mouse in the EAU (*n* = 6 mice) and control (*n* = 4 mice) group along with the standard deviation (error bars) was calculated and plotted against time. Progression of EAU in retinal tissue showed a sharp increase in weeks 2–4 followed by a step-like growth before reaching the maximum possible score in week 9. Significant differences between EAU and control group clinical scores were calculated using a multiple *t*-test. EAU mice had significantly higher clinical scores (** *p* < 0.05) from week 2 onwards. Control mice showed no signs of lesions or scarring in the retina throughout the 12-week period. (**c**) Progression of clinical scores in weeks 3–12 relative to the onset of changes in retinal tissue in week 2. The progression was found to be sigmoidal (r^2^ = 0.9873), highlighting a steady increase in clinical scores in the early stages of the disease which began to slow down from week 10 onwards, reaching a plateau towards the end of the study.

Early signs of retinal tissue changes were observed as distinct round lesions (white spots) which appeared independent of the areas of cuffing, away from blood vessels (Figure 4a; blue arrows). Longer linear lesions, which appeared as extensions of cuffing (Figure 4a; red arrows), were manifested later and were less prevalent than round lesions. In most EAU mice, these lesions were observed in small numbers within the first few weeks, resulting in lower clinical scores (Figure 4b), and were preceded by optic disc swelling, blood vessel swelling and cuffing. As explained in Table 1, the number of round and linear lesions present in all five fundus images determined the lower grade thresholds (Figure 4a; grade 1 and 2) following which the area of aggregated lesions and subsequent scarring became the more important factor for clinical scoring (Figure 4a; grades 3 and 4). As the disease progressed, the number and size of both round and linear lesions increased, resulting in higher clinical scores (Figure 4a; grade 3) in weeks 5–8 (Figure 4b). We again observed a step-like pattern in the disease progression though the plateau periods were shorter compared to those observed for the optic disc grading. By week 9, confluent large areas of aggregated round and linear lesions covering more than 10% of the fundus were observed (Figure 4a; white dotted line; grade 4) in 80–85% of the EAU mice (Section 3.5). These large areas of aggregated lesions led to scarring in all EAU mice and contributed to structural retinal damage, further explained below. In the following weeks, the area of scarring continued to increase; however, since the maximum possible score was already attained, clinical scores plateaued between weeks 10–12 (Figure 4c). As expected, clinical scores in EAU mice were significantly higher than those of control mice from week 2 onwards (Figure 4b, ** *p* < 0.05), a week later than the significance for optic disc and retinal blood vessel signs. The rate of progression showed a sigmoidal ascent from week 3 onwards, highlighting a steady rise in the scores before plateauing towards the end of the study period (Figure 4c).

#### 3.1.4. Slowly Progressing Structural Damage in the Retina Resulted in Moderately High Clinical Scores by Week 12

**Figure 5:**
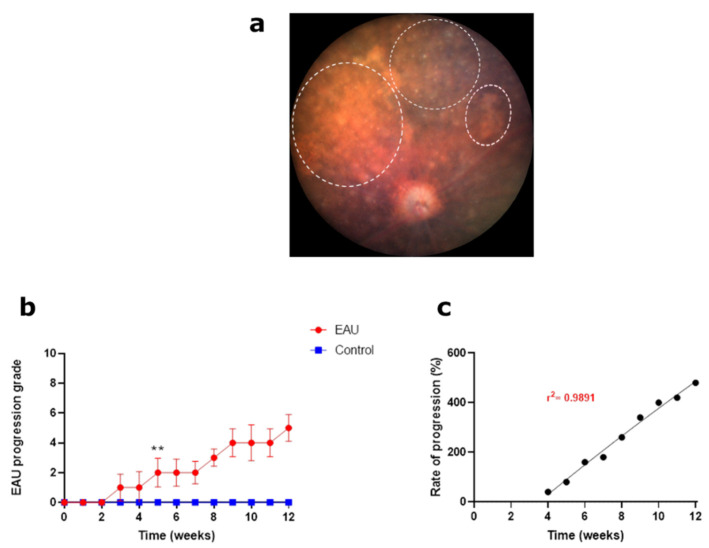
Signs of structural damage in the retina and clinical grading using fundus images. (**a**) Large areas of scarring seen in severe cases of retinal tissue damage which may be irreversible when EAU is resolved. No scarring; **grade 0.** More than 10 linear lesions or >10% region of scaring; **grade 1**. 10–25% region of scarring (white dotted line); **grade 2**. 25–50% region of scarring; **grade 3**. More than 50% region of scarring or retinal atrophy; **grade 4**. (**b**) Each eye was graded based on the pathologies observed in a set of five fundus images collected weekly for a period of 12 weeks. A weekly clinical score for retinal structural damage (maximum possible score of 8) was calculated for each mouse by adding the scores for the left and right eye. An average weekly score for each mouse in the EAU (*n* = 6 mice) and control (*n* = 4 mice) group along with the standard deviation (error bars) was calculated and was plotted against time. Progression of structural damage, tracked over a 12-week period, showed a step-like increase throughout the study period without reaching the maximum possible score indicating a lack of retinal atrophy. Significant differences between EAU and control group clinical scores were calculated using a multiple *t*-test. EAU mice had significantly higher clinical scores (** *p* < 0.05) from week 5 onwards. Control mice showed no signs of lesions or scarring in the retina throughout the 12-week period. (**c**) Progression of clinical scores in weeks 4–12 relative to the onset of structural damage in retinal tissue in week 3. The progression was found to be linear (r^2^ = 0.9891) highlighting a steady increase in structural damage severity.

In addition to highlighting the severity of EAU, certain retinal pathologies, such as linear lesions and large confluent areas of scarring also contributed towards the occurrence of structural damage in the retina (Table 1). In the initial stages, the small number of round lesions did not correspond to any retinal pathologies (as verified by OCT images discussed later) while linear lesions and large areas of scarring correlated with the presence of large granulomas, HRF and subretinal fluid (Section 3.4). Based on our observations (Figure 5a) and histopathological evidence seen in other studies [6], we concluded that areas of scarring correlated with structural damage of the retina, some of which may be permanent. In the first three weeks post immunisation, linear lesions were noted to be the most common sign of structural integrity loss in the retina (Figure A2d). Therefore, a small number of linear lesions, and later (weeks 3–5), a small area of scarring (0–25% of the total fundus area), contributed towards lower clinical scores (Figure 5a; grades 1 and 2). In the later stages, aggregated lesions covered large areas of the fundus (Figure 5a; white dotted lines), indicating more severe structural pathologies affecting multiple retinal layers. The progression of retinal scarring was very slow, reflected by longer periods of plateau in weeks 5–11 (Figure 5b). During these plateau periods, the area of retinal scars continued to grow but not sufficiently enough to cross the thresholds, justifying a higher clinical score (25–50% for grade 3). The highest structural damage score assigned in EAU mice was grade 3 (Figure 5b) and was only observed in 50% of eyes at the end of the study period (Section 3.5). Although not seen in our mice, large areas of scarring in later stages of EAU can result in retinal atrophy, including retinal detachment and haemorrhage as well as loss of retinal layers (grade 4) [3,16]. Manifestation of structural damage was slow, reflected in the delayed significance of the clinical scores in EAU mice (week 5 onwards) compared to the control group (Figure 5b, ** *p* <0.05). Even with delayed manifestation, structural damage progressed steadily, reflected in the liner progression of the clinical scores in weeks 4–12. As the maximum possible clinical score was not reached by the end of the study period, the progression was still linear at the 12-week mark (Figure 5c).

### 3.2. OCT Imaging and Grading

Each high-resolution fundus-guided OCT image clearly depicted multiple layers of the retina including the nerve fibre layer (NFL), ganglion cell layer (GCL), inner plexiform layer (IPL), inner nuclear layer (INL), outer plexiform layer (OPL), outer nuclear layer (ONL), photoreceptor layer (PRL), and retinal pigment epithelium (RPE) (Figure 1b). Since the vitreous, being a gel-like structure, appears transparent (no solid mass to reflect the scanning beam), it is depicted as a black space above the retina. OCT images were graded as described below. Similar to the fundus images, no pathologies were noted in the vitreous or retinal layers of control mice and hence all animals received a clinical score of 0 for each inflammatory element (Table 2).

### 3.3. Slowly Progressing Structural Damage in the Retina Resulted in Moderately High Clinical Scores by Week 12

**Figure 6:**
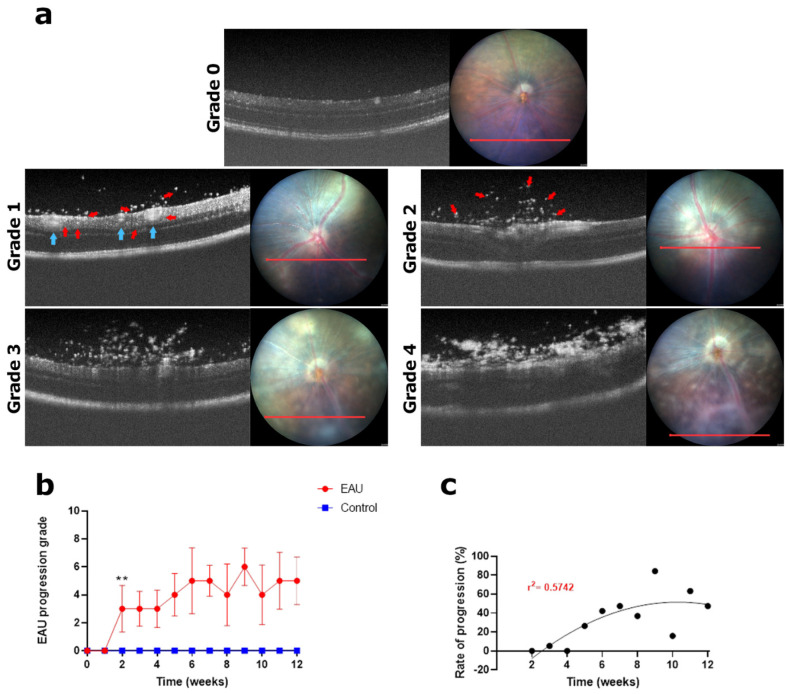
Signs of EAU in the vitreous and clinical grading using OCT imaging. The red line highlights the transverse fundus section corresponding to the OCT image. (**a**) Normal vitreous before inoculation which may or may not contain floaters (<20); **grade 0**. Infiltration of white specks into the vitreous (20–60); **grade 1**. Blue arrows indicate dilated blood vessels and red arrows indicate white specks in vitreous and retinal layers. Increase in the number of white specks in the vitreous (60–100); **grade 2**. Large, aggregated masses present in two to three OCT images; **grade 3**. Large dense aggregated masses present in four or more OCT images; **grade 4**. (**b**) Each eye was graded based on the pathologies observed in a set of nine vitreous-focused OCT images collected weekly for a period of 12 weeks. A weekly clinical score for vitreous health (maximum possible score of 8) was calculated for each mouse by adding the scores for the left and right eye. An average weekly score for each mouse in the EAU (*n* = 6 mice) and control (*n* = 4 mice) group along with the standard deviation (error bars) was calculated and plotted against time. Progression of changes in the vitreous, tracked over a period of 12 weeks, showed variable periods of clinical score increase with a high standard deviation indicating a fluctuation in infiltration within the EAU group. Significant differences between EAU and control group clinical scores were calculated using a multiple *t*–test. EAU mice had significantly higher clinical scores (** *p* < 0.05) from week 2 onwards. Control mice showed low numbers of white specks (<20) in the vitreous throughout the 12-week period. (**c**) Progression of clinical scores in weeks 2–12 relative to the onset of increasing numbers of white specks in week 1. The progression was found to be highly variable and could not be fitted to a particular trend (r^2^ = 0.5742).

**Figure A3:**
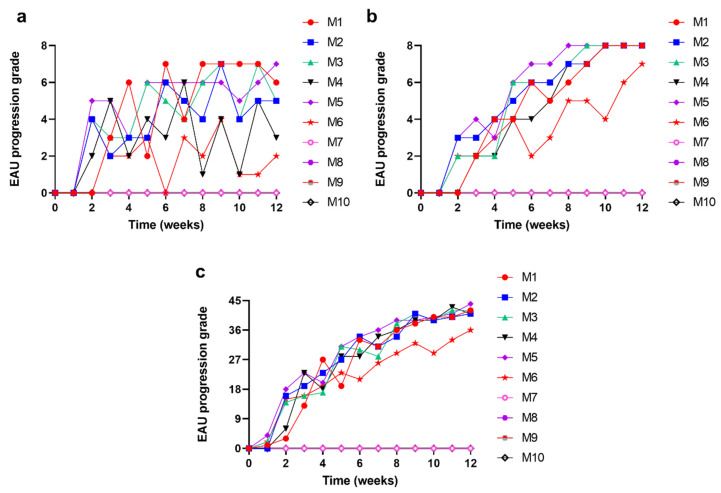
Clinical grading for each inflammatory element (i.e., vitreous and retinal layer) in each mouse using fundus image-guided OCT images and the total EAU response. Each eye was graded based on the pathologies observed in a set of nine images (nine with the vitreous and nine with the retinal layers in focus) collected weekly for a period of 12 weeks. The clinical scores for each mouse in the EAU (M1–M6) and control (M7–M10) group was plotted against time. (**a**) Progression of inflammation in the vitreous showed a high rate of variability in all EAU mice (M1–M6). There was significant leukocyte infiltration in some mice (e.g., M4) during some weeks before decreasing sharply the following week, highlighting the highly variable nature of the immune response. (**b**) Progression of inflammation in retinal layers showed a slow and steady increase in clinical scores throughout the study period with most EAU mice attaining the maximum possible score by week 10. As noted above, M6 had a slower inflammatory response and did not reach the maximum score by the end of the study period. (**c**) Progression of total EAU response showed a steady rise in all mice. The total EAU response score (maximum possible score of 48) was calculated per mouse per week by adding the scores from all six inflammatory elements. EAU response in all immunised mice increased steadily throughout the study period suggesting 100% EAU induction. All EAU mice, except for M6, showed a similar EAU response score (40–43) at the conclusion of the study. M6 depicted a slower growth curve with a final score of 36 in week 12. Control mice showed no signs of inflammation in the vitreous and retinal layers throughout the 12-week study period.

In OCT images, any form of infiltration into the vitreous manifested as bright white specks (small spots) in an otherwise black space above the retina. While some of the white specks were noted to be dispersed in the vitreous above the retina, others were attached to the NFL (Figure 6a; red arrows). Some specks were noted in both the control and EAU mice at the beginning of the study (day 0 before immunisation) which were concluded to be random ‘floaters’ (debris) occurring naturally. The entire vitreous of each eye was carefully scanned, and all specks were counted. The number of specks in each image was found to be less than 20. Following this observation, as stated in Table 2, we counted the number of specks in a set of nine vitreous images (one at the ONH, four superior and four inferior to the ONH) for each eye and assigned a clinical score of 1 if the sum of specks was between 20–60, marking the onset of EAU. In the first 1–2 weeks post immunisation, the rate of white specks appearance was very slow (Figure 6a; grade 1). Between weeks 2 and 4, there was an increase in the number of specks (Figure 6a; grade 2; 60–100) with some clumping together to form aggregates usually localised in two to three OCT images (Figure 6a; grade 3). White specks and denser clumps were also visualised in the corresponding fundus images coinciding with regions of cuffing and a general ‘hazy’ appearance of the retinal tissue. Some large clumps further developed into even denser masses (Figure 6a; grade 4) and were noted to be present in more than four OCT images obstructing the view of the retina in the corresponding fundus image. The level of white specks and clumps varied not only from mouse to mouse but was also distinct in each eye (one eye often had higher infiltration than the other), reflected by the higher standard deviation (Figure 6b). Although high numbers of specks were seen between weeks 3 and 9 in EAU mice, numbers were variable. We noted that the infiltration occurred in a ‘wave-like pattern’ greatly fluctuating from week to week in each animal (Figure A3a). When the average of the entire population was considered (*n* = 6 mice, 12 eyes), we observed an increase from weeks 4 to 7. Thereafter, clinical scores fluctuated between weeks 8 and 12, but remained high (>5 for both eyes combined) at the end of the study period (Figure 6b). Although there was high variability, the clinical scores in the EAU mice remained significantly higher than those of the control mice from week 2 onwards (Figure 6b, ** *p* < 0.05). However, the rate of progression did not follow a particular trend, as was evident from the low-curve fit score (Figure 6c; r^2^ = 0.5742).

### 3.4. Large Areas of Subretinal Oedema and Granulomas in Retinal Layers Led to the Highest Clinical Score by Week 11

**Figure 7:**
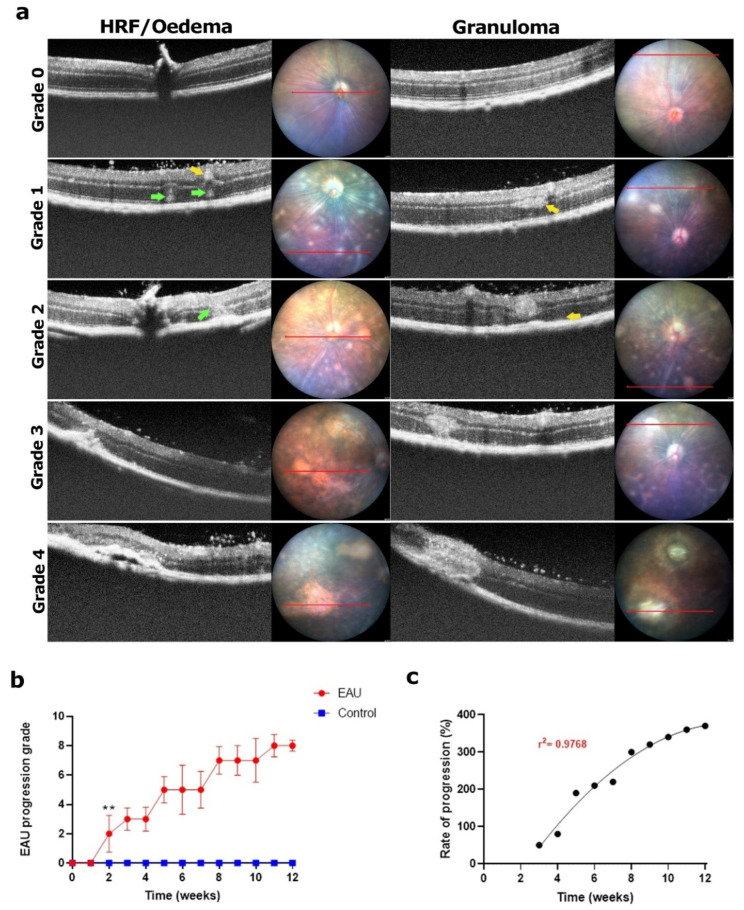
Signs of EAU in retinal layers and clinical grading using OCT images. The red line highlights the transverse fundus section corresponding to the OCT image. (**a**) Normal retinal layers with no pathology; **grade 0**. Development of HRF (green arrows; 5–10) in PRL and ONL or granulomas (yellow arrows; 5–10) in NFL, GCL, IPL and INL; **grade 1**. Two to five HRF advancing in size to disrupt the IPL and/or two to five granulomas enlarging to extend into the ONL; **grade 2**. More than two HRF advancing enough to disrupt multiple retinal layers and/or more than two granulomas enlarging to disrupt multiple layers; **grade 3**. HRF advancing to form more than two regions of oedema and/or granulomas enlarging in size and area to affect the entire retina; **grade 4**. Images in the left column depict HRF and granuloma advancement in line with EAU progression in the right column. (**b**) Each eye was graded based on the pathologies observed in a set of nine retinal layer-focused OCT images collected weekly for a period of 12 weeks. A weekly clinical score for retinal layer health (maximum possible score of 8) was calculated for each mouse by adding the scores for the left and right eye. An average weekly score for each mouse in the EAU (*n* = 6 mice) and control (*n* = 4 mice) group along with the standard deviation (error bars) was calculated and plotted against time. Progression of changes in retinal layers, tracked over a 12-week period, showed a step-like pattern before reaching the maximum clinical score possible in week 11. There was variability within the EAU group, as indicated by the error bars; however, by week 12, all mice showed signs of oedema and large granulomas. Significant differences between EAU and control group clinical scores were calculated using a multiple *t*-test. EAU mice had significantly higher clinical scores (** *p* < 0.05) from week 2 onwards. Control mice showed no signs of HRF or granulomas in retinal layers throughout the 12-week period. (**c**) Progression of clinical scores in weeks 3–12 relative to the onset of any signs of HRF or granulomas in week 2. The progression was found to be variable in weeks 3–7 following which it showed a steady rise trending towards plateauing by the end of the study period (r^2^ = 0.9768).

Early signs of disease manifestation included optic disc and blood vessel swelling followed by retinal tissue lesions and an increase in the number of white specks in the vitreous. In OCT images, we observed dilated blood vessels (Figure 6a; blue arrows) with white specks present in the surrounding retinal layers (Figure 6a; red arrows). These specks then clumped together to form masses presenting as ‘retinal folds’ or HRF (5–10) in the PRL and ONL (Figure 7a; green arrows) as well as ‘solid masses’ which indicate the presence of granulomas (5–10) in the NFL, GCL, IPL and INL (Figure 7a; yellow arrows) in EAU mice within 2–4 weeks post immunisation (Figure 7b, grade 1). As stated in Table 2, clinical scores in retinal layers depended on the severity and size of HRF and granulomas. As the disease progressed between weeks 4 and 8, formation of new HRF and granulomas was noted while the existing HRF increased in size to disrupt the INL (Figure 7a, green arrows) and granulomas increased in size to disrupt the ONL (Figure 7a; yellow arrows; grade 2). In the following weeks, HRF and granulomas increased in size to disrupt multiple layers but at a slower rate as depicted by longer periods of plateau in the clinical scores between weeks 5 and 12 (Figure 7a; grade 3). The rate of growth was also noted to be variable in EAU mice as highlighted by the larger error bars in Figure 7b. Towards the end of the observation period (weeks 9–12), HRF accumulated to develop into large empty vacuoles which indicate the presence of large sub-retinal fluid deposits (clinically termed oedema) and granulomas further increased in size covering almost the entire retina (Figure 7a; grade 4). This development was noted in 80–85% of the EAU mice (Section 3.5 and Figure A3b), resulting in the highest clinical score by week 11 (Figure 7b). We also observed that HRF and granulomas seen in OCT images correlated with regions of ‘cuffing’ or retinal lesions (round or linear) and in later weeks with large areas of scarring in the fundus images (Figure 7a). The mean clinical scores were found to be significantly higher in EAU mice compared to control mice from week 2 onwards (Figure 7b, ** *p* < 0.05). The rate of progression of the clinical scores, between weeks 3 and 12, relative to the onset of signs in retinal layers (week 2), showed high variability in the earlier stages due to the slow development of the pathologies. However, clinical scores progressively increased and plateaued, giving rise to a sigmoidal curve fit (r^2^ = 0.9768) by the end of the study period when all the EAU mice showed signs of either large granulomas or multiple regions of oedema or both (Figure 7c).

### 3.5. Slow and Steady Trajectory of EAU Signs over the 12-Week Study Period

**Figure 8:**
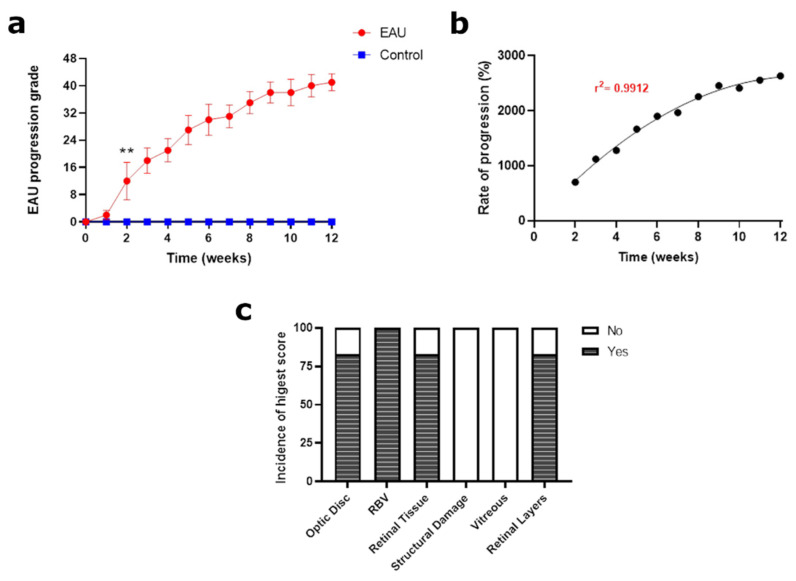
Total EAU response over the 12-week study period. (**a**) Grading of total EAU response combining the clinical scores from all inflammatory elements (i.e., optic disc, retinal blood vessels, retinal tissue, structural damage, vitreous and retinal layers). The clinical score from each inflammatory element for each mouse was added to obtain the total EAU response score (maximum possible score of 48) per mouse per week. An average weekly score for each mouse in the EAU (*n* = 6 mice) and control (*n* = 4 mice) group along with the standard deviation (error bars) was calculated and plotted against time. Progression of EAU, tracked over a 12-week period, showed a steady increase with very low variability (small standard deviation) indicating a uniform incidence and severity of inflammation in mice immunised with IRBP_1–20_ and PTX. Significant differences between EAU and control group clinical scores were calculated using a multiple *t*-test. EAU mice had significantly higher clinical scores (** *p* < 0.05) from week 2 onwards. Control mice showed no signs of EAU in any inflammatory element throughout the 12-week period. (**b**) Progression of clinical scores in weeks 2–12 relative to the onset of any EAU signs in week 1. The progression was found to be uniform with a steady rise trending towards plateauing by the end of the study period (r^2^ = 0.9912). (**c**) Incidence of highest (maximum possible) clinical score in EAU mice calculated at the 12-week mark. While 100% of EAU mice attained the maximum score of 8 for retinal blood vessels (RBV), only 80–85% EAU mice received the maximum score for optic disc, retinal tissue and retinal layers. None of the EAU mice obtained the maximum clinical score for structural damage and vitreous infiltrates due to slow development and high variability, respectively.

After calculating the clinical scores for each of the six inflammatory elements, the total EAU response was determined for each mouse and each week by adding up all six individual scores (maximum possible score 8 × 6 = 48; Figure A3c). The total EAU scores of the six EAU mice were then averaged per week to obtain a mean weekly EAU score which was then plotted against time including the standard deviation (depicted by the error bars) to track the progression of EAU over the 12-week period (Figure 8a). Upon analysis of the total EAU responses, we noted a sharp increase between weeks 1 and 2, indicating the onset of EAU, followed by a steadier increase between weeks 2 and 12. Error bars were within an acceptable range, confirming that EAU expression was relatively uniform, although clinical scores for individual inflammatory elements were sometimes variable. By the end of the study period, EAU scores were still increasing and below the maximum possible score of 48. However, the rise in clinical scores slowed down in the last weeks and we observed a trend towards a possible plateauing of the EAU response. The steady rise in EAU response is also justified by the rate of progression of the clinical scores between weeks 2 and 12 relative to the onset of EAU in week 1 (Figure 8b). The progression of clinical scores adopted a sigmoidal curve fit with a sharp trajectory indicating the uniform development of EAU with the progression slowing down by the end of the study period with a maximum possible score attained by 85–90% of the mice in four inflammatory elements (Figure 8c).

## 4. Discussion

**Figure A4:**
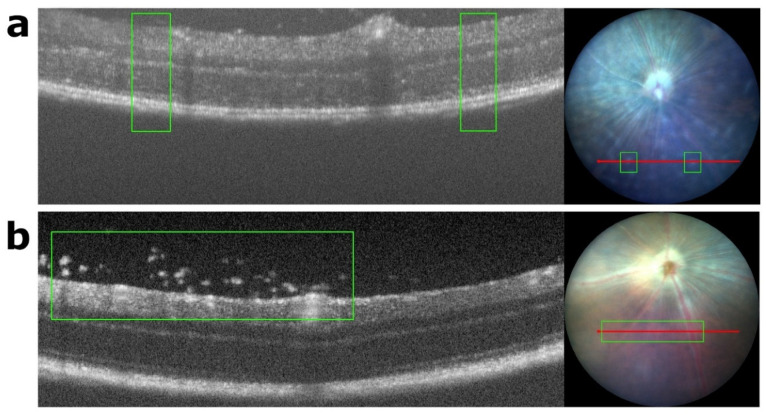
Comparison of presence of small focal lesions in fundus and OCT images. The red line highlights the transverse fundus section corresponding to the OCT image. (**a**) Depicts the presence of small round focal lesions in fundus images (green square) which do not correspond to any pathology observed in the retinal layers in the associated OCT image (green rectangles). This suggests that smaller round lesions appearing in the initial stages of EAU development may not affect the retina. (**b**) Depicts the presence of white specks (immune cells) in the vitreous in an OCT image (green rectangle) which is not visible in the corresponding fundus image (green rectangle), suggesting that some early signs of uveitis are not accurately represented in a fundus image.

Clinical grading of EAU using fundoscopy images has already been established [14] and has been used in multiple studies [26,27]. However, grading using fundoscopy alone lacks the visualisation of some pathologies which may require a cross-sectional view of the retina using OCT [16,23]. At the onset of EAU, we observed multiple changes in the eye which were caused by inflammation as a result of the immune response to human IRBP_1–20_ and PTX. These changes included optic disc swelling [28], blood vessel swelling, referred to as vasculitis in previous studies, followed by cuffing caused by the perivascular infiltration of leukocytes [3] as well as lesions and scarring of the retinal tissue also caused by immune cells [10]. These clinical uveitis signs, also observed in fundus images in humans, worsen as the disease progresses and may result in blindness [8]. Although not graded in humans, the accurate assessment of these signs and tracking of their progression is crucial when evaluating novel therapies in preclinical studies. In our study, for the first time, we developed an improved fundus grading system by assigning quantitative parameters to retinal pathologies such as the area of lesions in the optic disc or the area of scarring in retinal tissue. This reduces ambiguity between observers with the assigned clinical scores useful to quantitatively assess the various inflammatory elements. However, even with these quantitative thresholds, fundus images alone provide only a partial picture of the inflammatory changes occurring in uveitis. For instance, there is generally a breakdown of the blood–retinal barrier and increased adhesion of inflammatory cells in retinal blood vessels before any obvious signs of immune cell infiltration in the fundus which can be very mild at the beginning [29]. Furthermore, certain pathologies, such as small round focal lesions which appear in the early stages of EAU in fundus images, are not visible in retinal layers in the corresponding OCT images while infiltrating immune cells in the vitreous (early stages of vitritis) are clearly visible in OCT images but are not apparent in the corresponding fundus images (Figure A4). As such, the accurate visualisation of these early signs of inflammation requires the use of both fundus and OCT technologies. In addition, the progression of retinal atrophy, highlighted by loss of retinal layers and retinal detachment, cannot be accurately tracked based on fundus images and H&E staining (endpoint assessment). Therefore, an integrated system taking into account all the inflammatory elements seen in fundus and OCT images is necessary for assessing the severity of disease progression, as evident from our study.

The use of OCT imaging in inflammatory eye conditions is routine practice not only because of its diagnostic advantages but also because high-resolution SD-OCT images can highlight even the smallest changes [30]. OCT images can be useful for the objective assessment of therapies and can provide predictive parameters for the prognosis and recovery of visual function in patients [30]. In contrast, the acquisition of a clear OCT image in mice is difficult and the technology is still not widely available. However, since the inception of OCT imaging in rats and mice [15], many imaging platforms have been developed that can provide both fundus and OCT images using a single piece of equipment. Using fundus image-guided OCT images, we were able to clearly visualise the vitreous and retinal layers and note the occurrence of dilated retinal blood vessels and white infiltrates in the vitreous (clinical term ‘vitritis’) and retinal layers (clinical term ‘retinitis’). These white infiltrates have been shown to be infiltrating immune cells, entering the immune-protected regions of the eye (vitreous and retinal layers) via ‘leaky’ blood vessels, as a result of the immune response caused by the injected antigens (IRBP_1–20_ and PTX) which has been established in multiple studies using both OCT imaging and H&E staining [31,32,33]. In addition, we have also seen major disruptions of the retinal layers by granulomas and large regions of oedema which have been shown to cause sight-threatening complications in humans including retinal detachment and atrophy [34,35]. Therefore, OCT imaging in EAU mice, the most widely used preclinical EAU model, should become routine practice to provide more accurate and clinically relevant data when accessing novel uveitis treatments.

The use of OCT imaging to evaluate retinal pathologies in an EAU model is not a new concept and has been successfully demonstrated in previous publications [16,20,36]. However, these studies did not clearly define thresholds to differentiate clinical grades and also did not account for the vitreous as a separate tissue affected by inflammation (clinical term ‘vitritis’), a major contributor to human uveitis [37]. The present study clearly established that the degree of vitritis is an important clinical sign for tracking the development and severity of the disease and demonstrates that ocular immune cell infiltration is not limited to the onset of uveitis but continues to occur throughout the disease without additional injections of IRBP_1–20_ and PTX (Figure 6). This highlights the cyclic nature of chronic uveitis represented in our EAU model, as seen in humans, with a relapse of inflammation occurring, although other uveitis signs may have resolved [6,33]. Therefore, grading the vitreous separately becomes an important factor in accurately tracking disease progression and treatment efficacy.

In addition, previous studies did not accurately account for the development of HRF and granulomas in retinal layers as depicted in OCT images in our study. While Harimoto et al. [16] identified retinal folds as granulomas (originating in the PRL and extending into the ONL), other studies have shown that retinal folds and granulomas are different pathologies [3,14,27]. In our study, we also noted the presence of dense granulomas (a mass of cells) manifesting in the INL (appearing as solid white masses in OCT images) separate from the cone-shaped retinal folds, classified as HRF, manifesting in the PRL. The presence of these HRF, worsening to form regions of oedema, has also been observed in other ocular inflammatory conditions such as diabetic retinopathy [24] lending further credibility to our approach of tracking the development of HRF (Figure 7a; green arrows) and granulomas (Figure 7a; yellow arrows) separately. We observed the development of HRF into sight-threatening large regions of oedema and small lesions into large granulomas (Figure 7a) affecting multiple retinal layers. Thus, we quantitatively assessed the progression of these pathologies and provided definitive thresholds for each grade (Table 2). Overall, our study used fundus image-guided OCT images to better depict inflammatory changes in vitreous and retinal layers and has provided quantitative and qualitative thresholds to facilitate grading and reduce ambiguity between observers.

Several recent studies have also attempted to provide a quantitative grading scale using automated three-dimensional cell counting in conjunction with algorithms for the measurement of retinal layer thickening, retinal artifacts (granulomas) and vasculature thickness as seen on OCT images [32,38]. These multimodal imaging approaches, though very accurate, require a high degree of optimisation and technical knowledge that may not be readily available to every lab. This is substantiated by the fact that several recent studies have only analysed fundus images with the established grading system for evaluating EAU progression in mouse models [26,39,40]. In contrast, our grading system is simple and effective at capturing and assessing multiple aspects of inflammation using a combination of fundus and OCT images, thus capturing even minor changes more accurately and in a quantitative fashion. This renders the method more sensitive compared to prior grading systems as it includes more specific grading points and has been developed in close collaboration with clinicians involved in the clinical assessment of human patients with uveitis. Moreover, combining fundus and OCT imaging allows for the monitoring of pathological changes in multiple regions of the eye and thus provides a more complete picture of disease progression. Nevertheless, further research is required to compare the new proposed grading system to the previous method and to validate it in different EAU models. Furthermore, the slow and steady increase in EAU response seen in our study is supported by Chen et al. [33], highlighting that the C57BL/6 EAU mouse model mimics signs of chronic uveitis seen in humans. All inflammatory signs seen in human endogenous uveitis are also present in our study animals except for macular oedema and degeneration as mice do not have a macula [1,33,34,35]. Overall, this confirms the importance of combining a reliable preclinical EAU model with a standardised grading system for the investigation of novel uveitis treatments.

Even with all these advantages, EAU induction in C57BL/6 mice is difficult with low induction rates (30–40%) and a fluctuating degree of inflammation often reported [41,42], although optimised IRBP and PTX doses have increased the EAU incidence (75–80%) in C57BL/6 mice [43,44]. Nevertheless, induction is often inconsistent, introducing ambiguity in any preclinical study utilizing this model. While there are negligible differences reported between males and females in terms of EAU induction rates, most studies have opted for female mice simply due to ease of housing [45]. In our pilot study, we faced similar challenges, with EAU induction rates being 80–85%, even with optimised IRBP_1–20_ and PTX doses. With further investigation, we gleaned that emulsion preparation was performed by simple extrusion with a pipette or syringe in most studies [3,46]. We observed that the emulsion was less stable when prepared by simple extrusion, with water and oil phases separating if left standing. Moreover, the viscosity of the emulsion was much lower when prepared by extrusion alone resulting in higher backflow when performing s.c. injections. This could potentially lead to an inconsistent dose being administered which would account for the variability in EAU induction. Using high-energy sonication to breakdown the oil phase into smaller droplets for even distribution within the water phase (nanoemulsion) is well documented [47]. Sonication has previously been suggested by Aggarwal et al. [3] to increase the viscosity of the IRBP and CFA emulsion and reduce air bubbles in the mixture. In our study, we sonicated the emulsion for 5 min post extrusion which not only increased its viscosity (and thus reduced backflow) but also produced a stable mixture without any phase separation even when left standing for extended periods of time. We believe that this resulted in a more consistent IRBP dose being administered, resulting in 100% EAU induction. Further study into the changing fluid dynamics of the emulsion is warranted to standardise EAU induction and increase reproducibility.

A limitation of the present study is the variability in the number of OCT images collected for each mouse eye as we tried to capture each pathology by scanning the entire eye. We selected nine images with the worst pathologies which could possibly result in bias; however, we assured that the selected images were adequately spaced so that the same pathology was not counted in more than one image. We carried out this process to record all pathologies as we were building a grading ‘tool’ that was inclusive of as many pathologies as possible. To correct this, we propose to further standardise the grading process for use in preclinical investigations by capturing each OCT image three steps (9 µm) apart along the longitudinal plane. This number was calculated by averaging the size of multiple small pathologies (both HRF and granulomas) to ensure no pathology was omitted (irrespective of its size) while still collecting a fixed number of images for each eye. The nine OCT image set (vitreous or retinal layer focused) would then be selected from the collected images to compare between control and study animals.

## 5. Conclusions

Combining fundus imaging with image-guided OCT enables the detailed visualisation of inflammatory signs seen in EAU and allows for the detailed tracking of each pathology over the entire study period. Combined with quantitative grading thresholds, we believe that the grading system developed in this study is a powerful and reliable tool to better assess disease progression as well as evaluate the efficacy of novel therapies. Ultimately, including imaging technologies and observing clinically relevant signs will improve the translatability of novel therapies from preclinical studies into the clinics.

## Figures and Tables

**Table 1:** Uveitis grading using fundus images. The grading is based on a set of five fundus images, captured with the ONH in five different positions (central, nasal, temporal, inferior and superior; Figure 1a), to cover the entire surface area of the eye. Each mouse is given a grade for optic disc, retinal blood vessel, retinal tissue condition and structural damage.

Grade	Optic Disc	Retinal Blood Vessels	Retinal Tissue	Structural Damage
0	No inflammation Margins of the optic disc clearly visible Blood vessels connecting to the ONH clearly visible	No inflammation Blood vessels (arteries and veins) not swollen (blood vessel dilation) No perivascular infiltration (‘cuffing’)	No inflammation No retinal lesions (white spots away from blood vessels—not part of cuffing)	Minimal inflammation Round focal lesions No scarring
1	Swelling of the optic disc Optic disc margin delineated (blurry) Blood vessels connected to ONH still clearly visible	Blood vessel swelling (usually veins) No cuffing Blood vessels clearly visible	Three to 10 small round retinal lesions (diameter usually smaller than blood vessels) and/or One to four linear lesions	Mild inflammatory damage More than 10 linear focal lesions and/or One to two small scars covering > 10% of all fundus images
2	Optic disc swelling Optic disc margin highly diffused (halo effect) Localized infiltrates (white lesions) covering <10% of the optic disc Retinal vessels around ONH blurred but visible	Blood vessel swelling Mild cuffing on one to three retinal blood vessels that are surrounded by white lesions on one or both sides Thickness of cuffing on both sides combined less than diameter of blood vessel Smaller blood vessels may be blurry but larger ones are clearly visible	11–30 small round lesions and/or Five to 10 linear lesions (often present near blood vessels but isolated from cuffing)	Moderate inflammatory damage Three to five scars with scarring area covering 10–25% of all fundus images
3	Optic disc margin not visible Inflammatory lesions covering 10–30% of optic disc More than 30% of retinal vessels blurred by lesions	Blood vessel swelling may be present or resolved Small perivascular infiltrates may combine to form larger cuffing areas around two to five larger blood vessels One to three small blood vessels obscured by cuffing Thickness of cuffing greater than diameter of larger blood vessel Large blood vessels (>50–60%) are blurry but visible	More than 30 small round lesions and/or More than 10 linear lesions and/or Small scarring areas covering 1–10% of fundus area	Moderate–severe inflammatory damage Large scars with scarring area covering 25–50% of all fundus images
4	More than 30% of ONH covered by lesions More than 30% of retinal vessels around ONH covered by lesions ONH atrophy indicated by ‘sunset’ halo around optic disc and glassy opaque appearance around ONH	Large areas of perivascular cuffing on more than four blood vessels Thickness of cuffing on one or both sides combined greater than diameter of larger blood vessels More than four larger blood vessels obscured either in one or multiple segments	Confluent large areas of aggregated round or linear lesions in >10% of fundus area and/or Retinal atrophy or loss of retinal layers and retinal detachment (described by Chen et al. [23])	Severe inflammatory damage Large scarring area covering > 50% of all fundus images Scars can grow to merge reducing the number but increasing the affected area

**Table 2:** Uveitis grading using OCT images. The grading is based on a set of nine OCT images; one with the ONH at the centre and four obtained at random positions superior and inferior to the ONH. Each OCT image position is indicated by a red line in the corresponding fundus image. Each eye is given a grade for vitreous and retinal layer condition. The final clinical OCT score for each mouse eye is a summation of the two grades. HRF: hyper-reflective foci.

Grade	Vitreous	Retinal Layers
0	No inflammation ‘Floaters’ (random debris) usually present in general population observed as ‘white specks’ Number of ‘floaters’ < 20	No inflammation No HRF/no granulomas
1	Presence of white specks, in the vitreous; vitritis Total number of white specks in all nine OCT images is 20–60	Five to ten small HRF in PRL and ONL but not affecting the INL and/or Five to ten small granulomas (mass of cells) in NFL, GCL, IPL and INL but not extending into the ONL
2	Total number of white specks in all nine OCT images is 60–100 One to two OCT images show small aggregates of leukocytes	Two to five HRF disrupting the IPL and/or Two to five existing granulomas increasing in size to disrupt the ONL Partial disruption of retinal layers
3	Large aggregates of white specks seen in two to three OCT images	More than two existing HRF increasing in size to disrupt multiple layers and/or More than two existing granulomas increasing in size to disrupt multiple retinal layers including the RPE and choroid
4	Large aggregates of white specks localized in four or more OCT images, located inferior, superior and on the ONH High degree of vitritis usually observed in the inferior region	Formation of more than two oedemas (fluid filled regions) from HRF in retinal layers and/or Granulomas enlarging to disrupt multiple retinal layers including the RPE and choroid and/or Disappearance of retinal layers, usually NFL, GCL or IPL [16,23]

## Data Availability

All data is contained within the article or Supplementary Material.

## Supplementary Materials

The following supporting information can be downloaded at: https://www.mdpi.com/article/10.3390/biomedicines11072022/s1, File S1: The ARRIVE guidelines 2.0: author checklist.
